# The Role and Potential of ^18^F-FDG PET/CT in Malignant Melanoma: Prognostication, Monitoring Response to Targeted and Immunotherapy, and Radiomics

**DOI:** 10.3390/diagnostics12040929

**Published:** 2022-04-08

**Authors:** Luca Filippi, Francesco Bianconi, Orazio Schillaci, Angela Spanu, Barbara Palumbo

**Affiliations:** 1 Nuclear Medicine Unit, “Santa Maria Goretti” Hospital, Via Antonio Canova, 04100 Latina, Italy; 2 Department of Engineering, Università Degli Studi di Perugia, Via Goffredo Duranti 93, 06135 Perugia, Italy; francesco.bianconi@unipg.it; 3 Department of Biomedicine and Prevention, University Tor Vergata, Viale Oxford 81, 00133 Rome, Italy; orazio.schillaci@uniroma2.it; 4 IRCCS Neuromed, 86077 Pozzilli, Italy; 5 Unit of Nuclear Medicine, Department of Medical, Surgical and Experimental Sciences, University of Sassari, Viale San Pietro 8, 07100 Sassari, Italy; aspanu@uniss.it; 6 Section of Nuclear Medicine and Health Physics, Department of Medicine and Surgery, Università Degli Studi di Perugia, Piazza Lucio Severi 1, 06132 Perugia, Italy; barbara.palumbo@unipg.it

**Keywords:** malignant melanoma, PET/CT, ^18^F-FDG, BRAF mutation, radiomics, artificial intelligence, precision medicine

## Abstract

Novel therapeutic approaches, consisting of immune check-point inhibitors (ICIs) and molecularly targeted therapy, have thoroughly changed the clinical management of malignant melanoma (MM), the most frequent and deadly skin cancer. Since only 30–40% of MM patients respond to ICIs, imaging biomarkers suitable for the pre-therapeutic stratification and response assessment are warmly welcome. In this scenario, positron emission computed tomography (PET/CT) with ^18^F-fluorodeoxyglucose (^18^F-FDG) has been successfully utilized for advanced MM staging and therapy response evaluation. Furthermore, several PET-derived parameters (SUVmax, MTV, TLG) were particularly impactful for the prognostic evaluation of patients submitted to targeted and immunotherapy. In this review, we performed a web-based and desktop research on the clinical applications of ^18^F-FDG PET/CT in MM, with a particular emphasis on the various metabolic criteria developed for interpreting PET/CT scan in patients undergoing immunotherapy or targeted therapy or a combination of both. Furthermore, the emerging role of radiomics, a quantitative approach to medical imaging applying analysis methodology derived by the field of artificial intelligence, was examined in the peculiar context, putting a particular emphasis on the potential of this discipline to support clinicians in the delicate process of building patient-tailored pathways of care.

## 1. Introduction

Malignant Melanoma (MM), deriving from the malignant transformation of melanocytes, is the most frequent and lethal form of skin cancer and one of the most relevant tumor-related causes of death worldwide [1]. It commonly arises from cutaneous melanocytes (cutaneous melanoma), while mucosal and uveal melanoma which arise, respectively, from melanocytes located in the mucous membranes and ocular stroma, are less frequent [2]. Aside the aforementioned MM subtypes, the so-called acral melanoma, localized in glabrous skin of the palms, soles and nail beds, also has to be cited. The incidence of MM has been reported to be increasing in recent decades, with a significant prevalence in fair-skinned populations [3]. 

The most common type of cutaneous melanoma is represented by superficial spreading melanoma (SSM), often characterized by a good prognosis due to its relatively low Breslow thickness [4]. Exposure to ultraviolet (UV) radiation, particularly UVA (315–400 nm) and UVB (280–315 nm), has been recognized as a major risk factor for MM development since UV induces the cytosine to thymine transitions (i.e., C > T) at pyrimidine levels, the most frequently detected mutation in chronically sun-damaged skin. 

The Clark model has been widely utilized to describe MM progression and metastatization process [5]. It foresees a linear pathway with several crucial steps, leading from the benign precursor lesion (melanocytic nevus) to the dysplastic nevus and then through progressive radial and vertical growth phases up to malignancy and metastasis. The whole process, described by the Clark model, is driven by the accumulation of genetic and epigenetic mutations [6].

One of the most characteristic features of MM is its biological microenviroment, constituted by both cellular and humoral elements. Scientific evidence indicates that MM tumor microenviroment (TME) is a dynamic rather than a static entity since its composition (extracellular matrix, soluble factors, stromal and immune cells, etc…) changes during the different steps leading from the precursor lesion to the metastatization state, according to tumor’s biological behavior and grade of aggressiveness. 

Of note, it has been reported that between 40% and 60% of MM cases present mutation in exon 15 of the v-RAF murine sarcoma viral oncogene homolog B (BRAF), leading to valine (V) changing into glutamic acid (E) as a result of substitution at this exon (GTG > GAG) in the second placement of codon 600 (V600E) of BRAF kinase [7], namely BRAF V600E mutation. Further to the aforementioned BRAF V600E, other mutations, although significantly less common, have been detected at the molecular analysis of MM specimen [8]. 

The BRAF gene encodes RAF proteins, which are serine/threonine kinases representing a crucial part of the MAPK pathway which is, in its turn, deeply involved in regulating cell proliferation, differentiation and survival. In their normal condition, RAF proteins are activated by extracellular signals binding to the G-protein coupled receptors expressed on cell membrane. Conversely, the aforementioned BRAF mutation leads to locked RAF into an active position thus constitutively resulting in a ten-fold increased signaling through MAPK cascade [9].

Both TME and BRAF-mutations represent valuable targets for novel therapeutic approaches in MM, such as immune checkpoint (ICIs) and BRAF-inhibitors, that have thoroughly changed MM management and prognosis. 

In this scenario, imaging biomarkers suitable for identifying patients who are more likely to benefit of a specific therapy are warmly welcome in order to help clinicians define the most appropriate therapies.

Positron emission computed tomography (PET/CT) with ^18^F-fluorodeoxyglucose (^18^F-FDG) has a well-established role in oncology, both for staging and monitoring response to therapy. In this paper, we cover the current role of ^18^F-FDG PET/CT in MM, with a special focus on the management of patients submitted to the various therapeutic regimens, also highlighting the potential of radiomics, a novel discipline aimed at providing a quantitation of textural information by applying analysis methodology derived by the field of artificial intelligence [10].

## 2. PET/CT with ^18^F-FDG for Prognostication and Staging of Malignant Melanoma

Since the early 1990s, nuclear medicine (NM) has represented a valuable imaging approach for MM management due to the implementation of lymphoscintigraphy (LS) for the lymphatic mapping and sentinel node biopsy (SNB) [11]. Over the years, the contribution of NM has gained more and more consideration due to several technological improvements such as the use of portable imaging devices aiding the intraoperative detection of SN in difficult anatomical sites [12,13,14] or the utilization of hybrid imaging through single photon computed tomography (SPECT/CT) for an accurate localization of the scintigraphic data [15,16,17]. Table 1 summarizes the main manuscripts focusing on the role of ^18^F-FDG PET/CT for the imaging of advanced MM.

As a radionuclide imaging modality, ^18^F-FDG PET/CT is widely used for tumor staging, prognostication, and assessment of therapy response, due to its capability to identify tumor tissue due to the increased consumption of radiolabeled glucose. However, PET/CT showed a low detection rate of MM distant metastases in patients at American Joint Committee on Cancer (AJCC) stage I and II, while it was more impactful in case of advanced MM [25]. In particular, ^18^F-FDG PET/CT was reported to be less sensitive with respect to LS for the detection of nodal metastases at the initial staging, therefore LS and SNB are recommended as NM standard procedure for staging and stratification for adjuvant therapy of patients with AJCC stage I-II.

According to the various international guidelines, the use of ^18^F-FDG for staging MM patients should be limited to the advanced stage or in case of doubtful findings at conventional imaging [25]. 

A meta-analysis carried out by Xing and coworkers [26] compared ultrasonography (US), computed tomography (CT) and PET/CT for the staging and monitoring of MM patients. While US was found the optimal imaging modality for node staging, PET/CT showed the highest sensitivity (80%) and specificity (87%) for the detection of distant metastases. 

This report substantially aligns with the findings from Schröer-Günther and colleagues, who performed a systematic review focused on the usefulness of ^18^F-FDG PET/CT for MM staging: the authors collected and analyzed 17 clinical studies encompassing patients at I-IV AJCC stage. PET/CT’s sensitivity ranged from 33% to 97%, while specificity from 56% to 98% [27]. The authors attributed this wide spectrum to the various technologies applied (emissive only, i.e., PET, versus hybrid emissive/transmissive, i.e., PET/CT) and also to the different studies’ indication (nodal versus distant metastases detection). Of note, at sub-group analysis PET/CT’s sensitivity and specificity was significantly higher (sensitivity 68–87%, specificity 92–98%, respectively) in MM patients at stage III and IV with respect to MM at an earlier stage. 

It has to be highlighted that one of the most relevant limitations of ^18^F-FDG PET/CT for the imaging of MM is represented by its low sensitivity to the detection of brain metastases due to the non-favorable tumor-to-background uptake ratio [28]; therefore, magnetic resonance imaging (MRI) represents the gold standard for the detection of cerebral lesions [29].

It is worth mentioning that ^18^F-FDG PET/CT was found to significantly impact the management of patients affected by advanced MM: Gulec et al. retrospectively evaluated clinical records of 59 patients affected by known or suspected metastases from MM, submitted to brain MRI, total body CT and ^18^F-FDG PET/CT. Clinical management was firstly assessed on the basis of MRI and CT results alone, then re-assessed after the incorporation of PET/CT’s findings [18]. In comparison to CT, ^18^F-FDG PET/CT detected a greater extent of disease in the 55% of examined subjects and significantly influenced the treatment plan in the 49% of cases. 

Holtkamp et al. [22] assessed PET/CT’s impact on clinical management of subjects with in-transit or satellite MM metastases (S&ITM) through a prospective clinical study: they enrolled 25 patients who underwent brain MRI and PET/CT. The treatment plan was determined on the assumption of no distant metastases and then revised after the incorporation of imaging results. In the examined cohort, no brain pathological lesions were disclosed by the MRI, while ^18^F-FDG PET/CT detected distant metastases in 4 cases (16%). Furthermore, in 10 (40%) of the included subjects PET/CT revealed the appearance of metastases during follow-up within 6 months from diagnosis. In light of the above, PET/CT should be recommended for the staging and monitoring of high-risk MM patients presenting S&ITM at diagnosis, with a follow-up interval ranging from 3 to 6 months. 

From a technical point of view, it has been debated whether or not scanning the lower limbs might represent an additional benefit, in comparison with conventional whole-body field of view (i.e., from proximal thigh to skull base) in patients affected by MM without known focuses involving lower limbs. In a retrospective study on a large cohort of patients (*n* = 122), an additional scan of the lower limbs did not reveal further pathological localizations and never impacted on patients’ management [19]. Therefore, unless MM cutaneous metastatization to the lower limbs is known or suspected, the authors suggest that conventional whole-body PET/CT scan is the most suitable approach. 

Another widely discussed issue is if the diagnostic performance of PET plus contrast enhanced CT (PET/ceCT) might be superior with respect to PET performed without contrast-enhanced CT (PET/CT): in a cohort of 50 patients Pfluger and coworkers found that the sensitivity of PET/ceCT and PET/CT resulted in 97% and 100%, respectively, while specificity resulted in 93% for both modalities. In light of the aforementioned results, the use of a conventional PET/CT (i.e., without contrast media administered at CT-session) approach might be considered adequate for the imaging of advanced MM [20]. 

The scientific data concerning the utilization of ^18^F-FDG PET/CT for staging or restaging of uveal and mucosal melanoma are relatively few. In a retrospective study including 11 subjects with uveal melanoma, submitted to ^18^F-FDG PET/CT with different indications (for example doubtful findings at conventional imaging, *n* = 3; exclusion of further organs involvement in patients with known hepatic metastases, *n* = 5; restaging after systemic or loco-regional therapies, *n* = 4), PET/CT identified MM localizations to liver (83%), bones (42%), lymph nodes (33%), lungs (17%) and adrenal glands (8%) [23].

Cohen and coworkers analyzed the impact of the combined use of ^18^F-FDG PET/CT plus abdominal US in a large cohort encompassing 108 patients with medium to large uveal melanoma [24]. Among these subjects, only 2 presented liver metastases and 1 exhibited hepatic and extrahepatic metastatization. ^18^F-FDG PET/CT missed hepatic metastasis in 1 case but correctly identified extrahepatic colonization. In the whole examined cohort, PET/CT detected an additional unknown primary malignancy in 10 cases (9%). 

Aside from qualitative image interpretation, it is well known that PET technology allows for the calculation of several quantitative parameters, including maximum and mean standardized uptake value (SUVmax and SUVmean) and the more recently introduced volumetric ones, such as metabolic tumor volume (MTV) and total lesion glycolysis (TLG); this last result was the product of MTV and SUVmean. Of note, both MTV and TLG were found to be strictly correlated with tumor aggressiveness and biological behavior and was impactful on patients’ prognosis [30,31,32].

The prognostic value of PET-derived parameters has been investigated in AJCC stage IIIB MM by Bastiaannet et al., who enrolled 80 patients bearing palpable, histology/cytology proven nodal MM metastases, submitted to ^18^F-FDG PET/CT before therapeutic lymph node dissection and then monitored for assessing disease free survival (DFS) [21]. The subjects were dichotomized using median SUVmax as a threshold in 2 groups classified as at low or high SUVmax, respectively. The authors found that, overall, 5-year DFS was significantly higher in patients with a low than in those with high SUVmax (i.e., 41% vs. 24%), thus confirming the prognostic impact of the aforementioned parameter for the pre-therapeutic stratification of patients. 

## 3. ^18^F-FDG PET/CT for Assessment of Response to Therapy

For many years, the therapy of advanced MM has been based on chemotherapy (i.e., dacarbazine) and interleukin (IL-2), both of which have provided an unsatisfying response rate and duration [33]. Therapeutic landscape of MM has been thoroughly revolutionized by the introduction of several immune-checkpoint blockers, approved for the treatment of patients with advanced MM, such as ipilimumab or nivolumab, and others. Concurrently, some drugs specifically targeting BRAF and MEK signaling pathways proved effective in MM patients harboring mutation of BRAF gene [34]. It has to be highlighted that, while in wild-type MM only immunotherapy can be used, in BRAF-mutated MM both targeted and immunotherapies are feasible, but which is their optimal sequence (targeted prior to immunotherapy or *viceversa*) is not completely understood [33].

^18^F-FDG PET/CT has been applied for monitoring MM patients in order to promptly discriminate responders versus non-responders to a specific therapeutic regimen. Of note, since immunotherapy and molecularly targeted therapies work through different mechanisms, several efforts have been made to develop PET-reading criteria suitable for each one of these therapeutic approaches. 

### 3.1. Targeted Therapy with BRAF-Inhibitors

A cornerstone in BRAF-mutated MM managements was the introduction in clinical practice of the ATP-competitive BRAF inhibitors, vemurafenib (PLX4032) and dabrafenib (GSK2118436), proved to determine high response rate in BRAF-mutated MM with meaningful impact on progression free survival (PFS) and overall survival (OS) respect to chemotherapy (i.e., dacarbazine) [34]. Since the introduction of molecularly targeted therapies in BRAF-mutated MM, ^18^F-FDG PET/CT scan has been applied for monitoring response and detecting the onset of acquired resistance (Table 2). 

In a first report from McArthur and colleagues, ^18^F-FDG PET/CT was applied for the early detection of response to the BRAF-inhibitor vemurafenib in 31 patients affected by advanced MM, previously tested as positive for BRAF-mutation through genetic analysis, who were submitted to PET/CT scan at baseline and 15 days after the start of targeted therapy [35]. Among the enrolled subjects, 24 were treated with 960 mg twice a day (i.e., maximum tolerated dose) and 4 received subtherapeutic dose. All of the subjects were monitored through ceCT and ^18^F-FDG PET/CT. As far as it concerns the CT part of the study, response to treatment was assessed according to RECIST criteria (Response Evaluation Criteria in Solid Tumors) [36], while for the PET/CT scan, response was defined by a 25% in SUVmax value, according to EORTC (European Organisation for Research and Treatment of Cancer criteria) [37]. Furthermore, the authors calculated the reduction in the percentage of injected dose (%ID) in all of the detected lesions comparing baseline with follow-up PET/CT scan. In all of the patients treated with potentially therapeutic dose, a metabolic response (partial or complete) was achieved and in 3 subjects, a complete metabolic response was obtained. The authors found a significant reduction in SUVmax and a decrease in the percentage of injected dose (%ID) in all identified disease sites, with positive correlation between decrease in SUVmax and %ID, thus suggesting a homogeneous response to BRAF-inhibitor among lesions within the same individual. Of note, the metabolic response correlated with a trend toward a longer PFS.

The intra-individual heterogeneity of response to BRAF-inhibitor (i.e., dabrafenib) was further investigated by Carlino et al. in 23 patients with BRAF-mutated MM, imaged through ^18^F-FDG PET/CT at baseline and after 15 days of targeted therapy [38]. In all of the enrolled patients, all of the ^18^F-FDG-avid lesions detected at baseline and follow-up PET/CT were analyzed and metabolic response was assessed according to the aforementioned EORTC criteria. The grade of metabolic heterogeneity was categorized as follows: (1) homogeneous response if >90% of lesions responded (complete or partial metabolic response) with no evidence of progressive disease; (2) heterogeneous response if some lesions responded but new lesions were evident at follow-up PET/CT or if >10% of lesions showed stable disease; (3) homogeneous non-response if no lesions achieved complete or partial response. The authors found homogenous metabolic response in 74% of cases, heterogeneous metabolic response in 26% of patients, while no subject showed homogeneous non-response. Of note, the homogeneous response to BRAF-inhibitor was correlated with a longer time to progression with respect to metabolically heterogeneous response (7.4 vs. 3.0 months). 

The prognostic impact of molecular response to targeted therapy was analyzed by Schmitt et al. in a retrospective analysis of 24 patients receiving a combination of BRAF and MEK inhibitors (dabrafenib plus trametinib) [39]. All of the subjects performed PET/CT scan at baseline and after an average time of 26 days of therapy: PET response was assessed according to EORTC criteria. The authors identified and measured SUVmax on all the ^18^F-FDG-avid lesions both at baseline and follow-up scan. Furthermore, two lesions were chosen for the implementation in a prognostic model: (a) the tumor with the highest SUVmax at baseline, and (b) the tumor with least change in SUVmax from baseline to follow-up. The authors found that change in SUVmax for the least responsive lesion was associated with PFS but not with OS.

Annovazzi and colleagues recently investigated the prognostic value of baseline PET-derived parameter MTV and metabolic response in 57 patients affected by BRAF-mutated melanoma and monitored through ^18^F-FDG PET/CT during anti BRAF/MEK treatment [40]. Metabolic response was assessed according to EORTC criteria: among the enrolled subjects, 34 were classified as responders and 23 as non-responders. Baseline high MTV (i.e., >56 cc) and more than 2 metastatic organs were associated with a shorter PFS. Through a multivariate analysis, after having categorized patients in different prognostic classes, a high MTV and lack of metabolic complete metabolic response resulted in independent predictors for OS.

The aforementioned cited papers [35,38,39,40] deserve further consideration. Firstly, all of the published studies strongly support the use of ^18^F-FDG PET in the clinical setting of BRAF-mutated MM submitted to targeted therapy, not only as a baseline examination for patients’ pre-therapeutic prognostic stratification but also for monitoring metabolic response to treatment. Secondly, in all cases, EORTC criteria were applied for defining metabolic response. In this regard, it has to be highlighted that EORTC have been widely utilized in clinical practice, although preliminary data suggest that the more recently introduced PET response criteria in solid tumors (PERCIST) might present a better correlation with patients’ outcome after therapy. Further studies, also examining larger cohorts through multicenter cooperation, are needed to define the most appropriate PET-reading criteria for the follow-up of MM patients during targeted therapy. 

**Figure 1:**
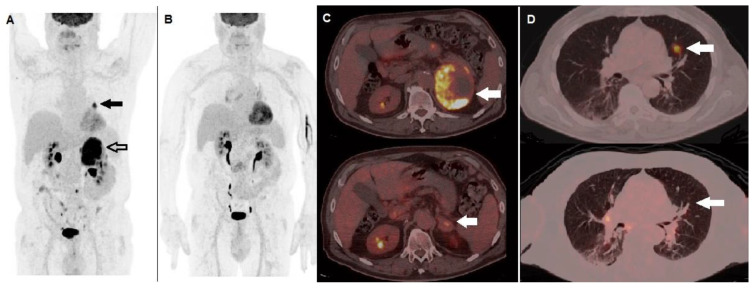
A 62-year-old male, previously submitted to excision of nodular cutaneous melanoma (Breslow thickness of 8 mm, Clark level IV, stage pT4a), performed ^18^F-FDG PET/CT for staging before therapy. (**A**) MIP image showed areas of increased tracer incorporation in the left lung (black arrow) and adrenal gland (black bordered arrow). Molecular analysis was positive for BRAF V600e mutation and he started combination of BRAF and MEK inhibitors (dabrafenib plus trametinib). PET/CT MIP (**B**) performed after 3 months showed metabolic response to therapy. Fused corresponding PET/CT axial of the abdominal region (**C**) demonstrated almost complete regression of the non-homogenously hypermetabolic lesion in the left adrenal gland when baseline (upper row, arrow) is compared with follow-up PET/CT scan (lower row, arrow). Fused PET/CT axial of the lung (**D**) demonstrated regression of the hyperactive nodule in the left lung when baseline (upper row, arrow) is compared with follow-up scan (lower row, arrow).

Figure 1 shows a case of BRAF-mutated MM patient, with a diffusely metastatic disease at staging, showing metabolic response at ^18^F-FDG PET/CT after anti BRAF/MEK therapy.

**Table 2:** Summary of main studies on ^18^F-FDG PET/CT for response assessment to targeted therapy in BRAF-mutated MM.

Authors	Year	Clinical Setting	N. of Patients	Applied Criteria	Comments
McArthur et al. [35]	2012	Part of a phase I clinical trial	*n* = 31	EORTC	^18^F-FDG PET/CT can be applied to assess response to vemurafenib; metabolic response is correlated with a trend towards a longer survival.
Carlino et al. [38]	2013	Part of a phase I clinical trial	*n* = 23	EORTC	Metabolic response to BRAF-inhibitor, categorized as homogeneous or heterogeneous through PET/CT, correlated with time-to-progression in patients with BRAF-mutated MM treated with dabrafenib.
Schmitt et al. [39]	2016	Retrospective, Single-center	*n* = 24	EORTC	In patients undergoing combined anti BRAF/MEK therapy, change in SUVmax for the least responsive site of disease resulted associated with progression free survival.
Annovazzi et al. [40].	2021	Retrospective, Single-center	*n* = 57	EORTC	Baseline MTV and complete metabolic response during BRAF/MEK therapy are predictors of OS in BRAF-mutated melanoma submitted to targeted therapy.

### 3.2. Immunotherapy: The Need for Novel Criteria 

Immunotherapy, termed as a cancer treatment based on unleashing host immune system to recognize and kill tumor cells, is not new in the history of medicine (i.e., interferon, cancer vaccines, etc.), but has been thoroughly revolutionized by the introduction of immune check-point inhibitors (ICIs) [41]. With respect to other immunotherapeutic approaches, ICIs, targeting specific co-inhibitory receptors involved in immune response regulation, proved to determine stronger and more prolonged effects [42,43]. 

The first immune-checkpoint blocker approved by the FDA and EMA for the treatment of advanced MM has been represented by ipilimumab, a monoclonal antibody (MoAb) directed towards CTLA-4 (Cytotoxic T-Lymphocyte Antigen 4), a member of immunoglobulin-related receptor, expressed on antigen-presenting cells (APC), and proved capable to prevent T cell activation [44]. Subsequently, other MoAbs have been developed, such as pembrolizumab and nivolumab, both directed towards programmed death protein-1 (PD-1), or atezolizumab, targeting the ligand of PD-1 (PDL-1). PD-1 was found overexpressed by activated T-cells, B-cells, dendritic cells, and natural killer cells, while PDL-1 is expressed by various tumor cells (melanoma, lung cancer, lymphoma, etc…). Of note, the binding between PD-1 and PDL-1, occurring at the TME level, results in a complex intracellular pathway, leading to the inhibition of T-cell functions, such as proliferation, cytotoxic activity, and the production of cytokines. 

**Figure 2:**
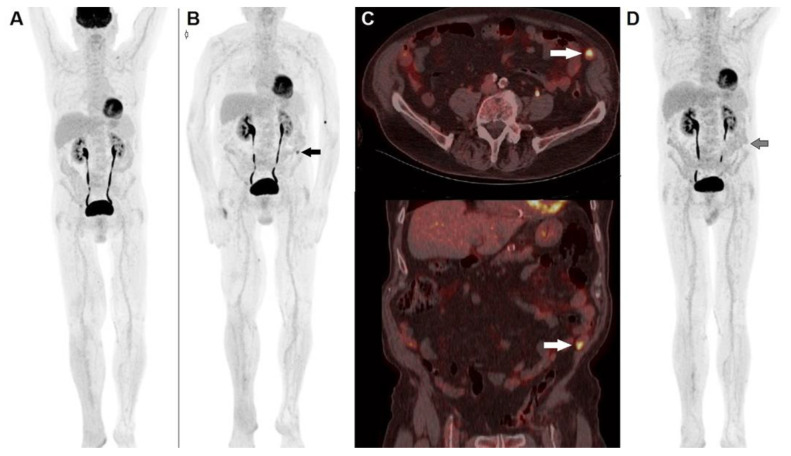
A 74-year-old male, previously submitted to excision of nodular cutaneous melanoma of the right foot (Breslow thickness of 8 mm, Clark level IV, stage pT4a), performed ^18^F-FDG PET/CT before the start adjuvant immunotherapy. (**A**) MIP image showed physiological tracer biodistribution, with no evidence of pathological accumulation. PET/CT MIP (**B**) performed after 3 months of PD-1 blocker (nivolumab) depicted the appearance of an area of increased tracer accumulation in the left iliac fossa (black arrow). (**C**) Fused corresponding PET/CT axial (upper row) and coronal (lower row) of the abdominal region demonstrated the onset of a hypermetabolic nodule next to the abdominal wall, suspected to be peritoneal localization (white arrow). The pattern was interpreted according to PERCIMT criteria (i.e., pseudo-progression) and the patient continued immune check-point inhibitor. A further PET/CT MIP (**D**) after 6 weeks demonstrated complete spontaneous regression of the area of increased ^18^F-FDG accumulation in the left iliac fossa (black arrow), thus confirming the diagnosis of pseudo-progression.

**Figure 3:**
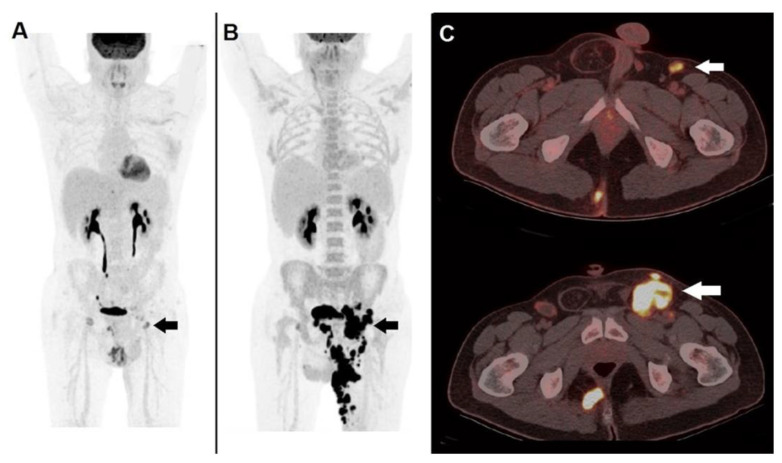
A case of hyper-progression during immunotherapy. A 41-year-old male, affected by non-melanoma skin cancer (locally advanced cutaneous squamous cell carcinoma) of the left thigh, submitted to anti PD-1 immunotherapy (cemiplimab). ^18^F-FDG PET/CT before the start of therapy: (**A**) MIP image showed pathological tracer accumulation within a left inguinal node (black arrow). PET/CT MIP (**B**) performed after 2 months of PD-1 blocker depicted an impressive disease progression at nodal and cutaneous level (black arrow). (**C**) Fused corresponding PET/CT axial (upper row) acquired before therapy well demonstrated a hypermetabolic inguinal node (white arrow), fused PET/CT after 2 months of PD-1 blocker showed meaningful enlargement of the metastatic node (white arrow). Immunotherapy was discontinued, the patient was switched to chemotherapy but deceased after 3 months.

The peculiar mechanisms by which ICIs work, that is, by removing an inhibitory signal, therefore enhancing immune system against tumor, entail some challenges in monitoring response to treatment. The enrollment of activated lymphocytes into tumor sites may produce two different patterns of apparent progression (i.e., pseudo-progression): (1) transitory dimensional increase in pre-existing lesions, followed by shrinking; (2) onset of new lesions, followed by a subsequent reduction in the overall tumor burden. In a recently published meta-analysis, the incidence of pseudo-progression in cancer patients treated with ICIs was reported to be 6–10%, more often occurring during therapy with ipilumumab [45]. Aside from pseudo-progression, another peculiar pattern of response to ICIs has been described, namely hyper-progression, represented by a massive, unexpected disease progression, correlated with a poor prognosis [46]. Figure 2 and Figure 3 show two distinct clinical cases of patients presenting pseudo-progression and hyper-progression during immunotherapy, respectively.

The complex pattern of response to ICIs has triggered the development of novel criteria, both for morphological (i.e., CT) and functional (PET/CT) imaging, with the aim of effectively monitoring patients during immunotherapy. Table 3 summarizes the main metabolic criteria applied for PET interpretation in cancer patients undergoing immunotherapy, while Table 4 illustrates the main manuscript focusing on the use of PET/CT for monitoring patients under ICIs treatment.

Sachpekidis et al. evaluated the usefulness of ^18^F-FDG PET/CT for monitoring patients with advanced MM submitted to immunotherapy with ipilimumab: every enrolled subject underwent PET/CT scan at baseline, after 2 cycles and after the end of treatment (4 cycles) [48]. Metabolic response was assessed according to EORTC criteria, and at the end of treatment, fifteen subjects were classified as having progressive metabolic disease (PMD), five as presenting stable metabolic disease (SMD) and two as showing partial metabolic response (PMR). “Interim” PET/CT scan after 2 cycles effectively predicted final metabolic response in 13 out of 15 subjects with PMD (86.6%), in all cases of SMD (100%) and in no case (0%) of responding patients (PMR). Both patients, finally categorized as having PMR, in fact showed pseudo-progression at early PET/CT and were, therefore, misclassified by EORTC criteria. It is worth mentioning that both early and late metabolic response strongly correlated with PFS and OS. The paper by Sachpekidis’s group strongly pointed out that metabolic response assessed by ^18^F-FDG PET/CT can predict survival benefit in patients treated with ipilimumab, nevertheless EORTC criteria might not be suitable for interpreting early PET/CT scan after 2 cycles of therapy due to the confounding effect of possible pseudo-progression. 

A further PET-interpreting approach, namely PERCIMT (PET Response Evaluation Criteria for Immunotherapy), was proposed by Anwar et al. [49], based on the number of newly emerged lesions during immunotherapy, rather than on changes in SUVmax. The authors retrospectively evaluated a cohort of 41 subjects with metastatic MM, submitted to ^18^F-FDG PET/CT scan before and after therapy with ipilimumab, monitored up to a median time of 21.4 months and dichotomized, on the basis of their clinical response, in patients with clinical benefit (CB) and without clinical benefit (no-CB). The group with CB encompassed patients presenting complete metabolic response (CMR), partial metabolic response (PMR) and stable metabolic disease (SMD), while the group with no-CB had progressive metabolic disease (PMD). The authors found that a threshold of 4 newly emerged ^18^F-FDG-avid lesions with functional diameter <1 cm on post-treatment PET/CT scan yielded a sensitivity of 84% and a specificity of 100% in predicting patients’ clinical outcome (CB vs. no-CB), while the cut-off resulted lower in case of newly appeared lesions with greater functional diameter. This novel PET-reading criteria was of particular interest; furthermore, they introduced the concept of “functional diameter” (i.e., diameter of newly emerged lesion measured on PET/CT images) to assess metabolic response during immunotherapy.

Goldfarb et al. developed a further possible approach for PET-interpretation during ICIs treatment, namely immune PET Response Evaluation Criteria (iPERCIST), that authors applied in subjects with lung cancer submitted to nivolumab [50]. In the aforementioned study, all of the patients had baseline PET/CT (SCAN-1) and were monitored with a further PET/CT after 2 months (SCAN-2): if progressive metabolic disease was detected at SCAN-2, they were categorized as having UPMD and were then scheduled to perform a further PET/CT (SCAN-3) after 4 weeks in order to exclude or confirm progression. To the best of our knowledge, iPERCIST criteria have not been specifically applied in MM submitted to immunotherapy yet, although they were found effective for monitoring subjects affected by locally advanced non-melanoma skin cancer submitted to PD-1 targeting immunotherapy [51].

The value of quantitative analysis in MM patients undergoing therapy with ipilimumab was assessed by Sachpekidis et al. [53] in a cohort of 25 subjects. Dynamic PET/CT (dPET/CT) of the thorax and upper abdomen as well as the static, whole-body PET/CT scan were carried out before treatment (baseline), after 2 cycles of treatment (interim PET/CT) and at the end of therapy (late PET/CT). Quantitative analysis of dPET/CT, including SUV, two-tissue compartment and fractal analysis, was performed and metabolic response was assessed according to PERCIMT criteria: both quantitative analysis and PERCIMT were correlated with clinical outcome (CB or no-CB). At final analysis, only PERCIMT criteria significantly predicted subjects’ benefit from immunotherapy. 

Ito et al. applied an immunotherapy-modified version of PERCIST, namely imPERCIST5 [52], for assessing the response to treatment in 60 patients submitted to ^18^F-FDG PET/CT before and after ipilimumab. Change in the sum of SULpeak (SUV normalized to lean body mass) of up to 5 lesions was gauged between baseline and follow-up PET/CT scan: with respect to traditional PERCIST, the appearance of new lesions did not entail the classification of PMD, but PMD was defined only by an increase in the sum of SULpeak by at least 30%. Of note, imPERCIST5 criteria significantly predicted patients’ 2-year OS and newly emerged lesions more commonly resulted in subjects with PMD than in responders (PMR+SMD). Partially in agreement with the report by Anwar et al. [49], the authors noted that in patients with PMR a number raging 2–4 lesions spontaneously regressed during the course of therapy. 

The combined use of immunotherapy and molecularly targeted therapy in BRAF-mutated MM may entail further challenges in assessing the response to treatment [54]. In this regard, it has to be reported the study by Sachpekidis et al. who evaluated through PET/CT longitudinal studies 15 patients undergoing combined regimen (vemurafenib plus ipilimumab). The authors compared two distinct criteria for response assessment: EORTC and PERCIMT. While EORTC correctly categorized 13 out of 16 patients, PERCIMT effectively discriminated patients with CB from those with no-CB in 15 out of 16 cases. Furthermore, the authors highlighted the usefulness of ^18^F-FDG PET/CT to promptly identify immune-related adverse events (irAEs) in 7 subjects. It has to be highlighted, in fact, that the stimulation of the immune system induced by ICIs can determine the onset of a wide spectrum of immune-mediated alterations of healthy organs (hypophysis, colon, skin, adrenal glands, etc…), namely irAEs, whose severe and potentially fatal consequences may require corticosteroid treatment and, in some cases, ICIs discontinuation [55].

The incidence and severity of irAEs has been reported to vary with the duration and the type of immunotherapeutic regimen, being more frequently observed with ipilimumab or in case of combined approaches [56]: in particular, the incidence of grade 3–4 irAES resulted in 15% during CTLA-4 targeted treatment and 5–6% when PD-1/PDL-1 blockers are utilized. 

**Figure 4:**
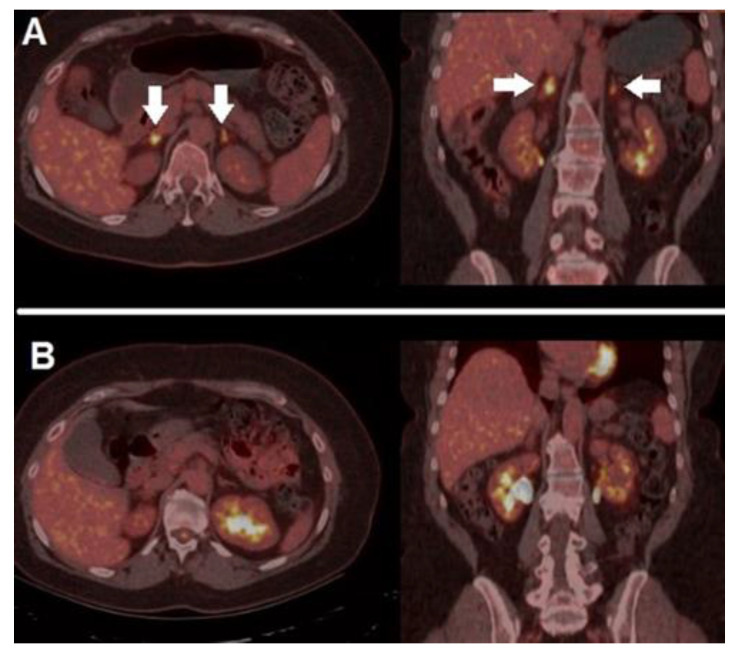
A case of irAEs. A 41-year-old female, previously submitted to excision of a nodular, high-risk MM, started adjuvant immunotherapy with nivolumab. After 4 cycles, she presented mild fatigue and grade 1 hyponatremia and orthostatic hypotension, with reduced level of morning cortisol. ^18^F-FDG PET/CT performed after symptoms’ onset (**A**) demonstrated bilateral increased tracer accumulation within adrenal glands (white arrows), compatible with the suspicion of immune-related adrenalitis, she discontinued immunotherapy and started on steroid therapy with excellent clinical response. PET/CT, acquired after steroid therapy and symptoms’ regression (**B**), revealed complete regression of the adrenal glands’ hypermetabolism. After multidisciplinary consensus meeting, immunotherapy was restarted, and no further complications were registered.

In a cohort of 290 subjects submitted to ICIs, Fujii et al. found irAEs (any grade) in 34% of cases, enterocolitis and dermatitis being the most common clinical manifestations [56]. Of note, the onset of irAEs, being strictly correlated with immune system stimulation and response, has been found to be associated with an improved overall response rate and a longer progression free survival. Figure 4 depicts a case of irAE (adrenalitis), which promptly regressed after corticosteroid treatment, in a MM patient treated with a PD-1 blocker.

Tumor metabolic heterogeneity has been reported as a relevant feature associated with response to ICIs [57]. In a retrospective study including 34 patients with MM treated with immunotherapy as a first (*n* = 23) or second (*n* = 11) line, Sanli’s group measured several PET-parameters (i.e., SUVmax, SUVpeak, MTV and TLG), also calculating tumor metabolic heterogeneity (TH) index, and correlated all these factors with patients’ outcome (OS and PFS). The authors found that TH index was inversely correlated with SUVmax and TLG; furthermore, a high TH index was a favorable prognosticator of OS. As the same authors state, TH arises as a result of various genetic, epigenetic changes in tumor during its growth and proliferation, as a consequence of therapy response and also as a response to environment modifications (e.g., changes in tumor oxygenation). Although preliminary studies highlighted the potential of TH index as a prognostic factor in oncology, several issues, particularly the various and not-standardized technical methodologies utilized for its calculation, have limited the deepening of the aforementioned parameter [58].

Nobashi and colleagues investigated the role of lymphoid-rich organs’ activation during treatment with ICIs for the prediction of response in 41 oncological patients, 21 of whom were affected by MM [59]. Changes in SUVmax were calculated for tumor, spleen, bone marrow, thyroid, and pituitary gland on baseline and on PET/CT scan at first restaging. Results were correlated with patients’ best overall response (BOR) assessed at 1 year after therapy commencement. The authors found that a decrease in the tumor’s SUVmax was associated with BOR and an increase in SUVmax of 1.5 or more in thyroid, reflecting immune system activation, was correlated with complete metabolic response. 

A report by Seban and colleagues further evaluated the prognostic role of PET-parameters and lymphoid organs’ metabolic activation in a retrospective study including 55 patients submitted to ^18^F-FDG PET/CT before PD-1 targeting immunotherapy. Among various parameters extracted from baseline PET/CT, high MTV, increased spleen and bone marrow-to-liver ratio (SLR and BLR, respectively) were significant predictors of survival in multivariate analysis [60]. It is worth mentioning that the authors also correlated PET/CT’s results with transcriptome analysis carried out on MM specimens through nanostring technology. Increased metabolic activity in lymphoid organs (spleen, bone marrow) was associated with the expression of a transcriptomic profile involving regulatory T-cell biomarkers, thus suggesting that, in some cases, a complex cross-talk between tumor and hematopoietic organs might reprogram hematopoiesis towards the production of immune-suppressive cells (e.g., tumor-associated macrophages, regulatory T-cells, myeloid-derived suppressor cells), thus favoring inflammatory response and tumor progression [61]. 

As far PET-volumetric parameters are concerned, Nakamoto reported that an MTV reduction at first restaging PET/CT scan, obtained after 3–4 cycles of immunotherapy, significantly impacted the patients’ outcome, since subjects with post-treatment low MTV had significantly longer OS than those with high post-treatment MTV [62]. 

It is still debated which of the aforementioned criteria (i.e., EORTC, PERCIST, imPERCIST5, iPERCIST, PERCIMT, changes in MTV or TLG, etc.) might be the optimal PET-interpreting approach in cancer patients treated with ICIs. In this regard, Annovazzi and colleagues [63] compared, in a retrospective study encompassing 57 MM patients, several CT-based and metabolic criteria (i.e., RECIST 1.1, EORTC, PERCIMT, changes in MTV and TLG) for assessing response to immunotherapy. Most interestingly, the authors found that a combination of PERCIMT and MTV criteria was the best predictor for subjects submitted to anti CTLA-4 therapy, while EORTC, TLG and MTV yielded the best results in patients treated with anti PD-1 drugs. These preliminary data suggest that a combination of different criteria, also taking into account the specific immunotherapeutic regimen, might be the best methodological approach for PET-interpretation in subjects submitted to ICIs.

Since the risk of irAEs onset has been reported to be associated with immunotherapy duration, it is crucial to determine when treatment should be discontinued. In this regard, Schank and collaborators [64] retrospectively analyzed 45 patients submitted to ICIs, who, after the execution of a PET/CT scan, discontinued immunotherapy due to either patients’ decision or irAEs evidence. The median time duration of immunotherapy before discontinuation was 21 months, at PET/CT carried out before therapy-discontinuation 32 subjects showed complete metabolic response (CMR) while the remaining 13 had no-CMR. Of note, at follow-up (median 34 months) 6 out of 13 no-CMR patients had progressive disease, while only 3 of 32 CMR-patients progressed. These preliminary results suggest that achieving CMR during immunotherapy represents a favorable predictive factor of outcome, even in case of treatment discontinuation.

Aligning with the report from Schank’s group [64], a recently published analysis performed by Dimitriou and colleagues [65] addressed the importance of metabolic response to ICIs, particularly in case of CMR, as a prognostic factor on sustained response after treatment discontinuation. The authors, in fact, found a 5-year PFS rate significantly higher in patients with CMR with respect to those with no-CMR. Furthermore, response assessed by metabolic criteria (EORTC) outperformed morphological assessment (RECIST) for the prediction of long-term outcome. Recently published reports from different groups have further underlined the high prognostic impact of complete metabolic response on long-term disease control after ICIs discontinuation [66,67]. In particular, a recent analysis of a large cohort (*n* = 140) evaluated the prognostic impact of PET-assessment before ICIs discontinuation on final outcome (i.e., melanoma-specific survival/MSS) by dichotomizing enrolled subjects in 2 groups: the “elective” group (ICIs discontinued due to clinical decision) and “toxicity” group (ICIs discontinued due to irAEs). At 29.3 months of median follow-up, the “elective” group contained a higher proportion of survivors and presented a significantly higher MSS as compared to “toxicity” group. Furthermore, an absence of ^18^F-FDG-avid lesions at the time of immunotherapy discontinuation resulted in a powerful predictor of post-treatment survival (MSS).

**Table 4:** Summary of main studies on ^18^F-FDG PET/CT for response assessment to immunotherapy with ICIs in MM.

Authors	Year	Therapy	N. of Patients	Applied Criteria	Comments
Sachpekidis et al. [48]	2015	Ipilumumab	*n* = 22	EORTC	Metabolic response to ipilimumab assessed by PET/CT correlates with survival benefit. EORTC might erroneously classify patients presenting pseudo-progression at early (i.e., after 2 cycles) PET/CT scan.
Anwar et al. [49]	2018	Ipilumumab	*n* = 41	PERCIMT	The number of newly emerged lesions and their functional diameter resulted significant predictors of patients’ outcome (clinical versus no-clinical benefit).
Sachpekidis et al. [53]	2018	Ipilumumab	*n* = 25	PERCIMT vs Quantitative analysis of dynamic PET/CT	Analysis of dynamic PET/CT acquired at different time-points (baseline, after 2 cycles and after 4 cycles) does not correlated with final outcome after immunotherapy
Ito et al. [52]	2019	Ipilumumab	*n* = 60	imPERCIST5	Change in SULpeak, measured in up to 5 lesions, between baseline and post-treatment scan meaningfully predicts the outcome after immunotherapy.
Sachpekidis et al. [57]	2019	Vemurafenib plus ipilimumab	*n* = 25	EORTC vs PERCIMT	PERCIMT outperformed EORTC criteria for assessing response to combined targeted therapy and immunotherapy in BRAF-mutated MM.
Sanli et al. [57]	2019	Ipilimumab, nivolumab	*n* = 34	PET-parameters correlation with OS	Tumor heterogeneity (TH) index was inversely correlated with SUVmax, SUVpeak, TLG and MTV, while it was a meaningful predictor of patients’ survival.
Nobashi et al. [59]	2019	Ipilimumab, pembrolizumab, nivolumab	*n* = 41	Correlation with BOR (best overall response)	Patients responding to immunotherapy showed a decrease in tumors’ SUVmax between baseline and follow-up scan, while complete response was associated with increased SUVmax in thyroid.
Seban et al. [60]	2019	PD-1 bockers	*n* = 55	PET-parameters correlation with survival	High MTV and activation of lymphoid organs at baseline represent unfavorable prognostic factors in patients undergoing anti PD-1 therapy.
Iravani et al. [68]	2020	Nivolumab plus ipilimumab	*n* = 31	PERCIST	Metabolic response correlates with survival benefit in patients submitted to ICIs. PET/CT scan is a valuable tool for the image of irAEs.
Annovazzi et al. [63]	2020	Nivolumab or ipilumumab	*n* = 57	PERCIMT, EORTC, RECIST 1.1, TLG, MTV	For patients treated with CTLA-4 targeting immunotherapy a combination of PERCIMT and MTV resulted the best approach for response assessment. In case of anti PD-1 therapy, the best results were achieved with EORTC, MTV and TLG.
Schank et al. [64]	2021	Nivolumab, pembrolizumab, ipilumumab	*n* = 45	EORTC, PERCIMT	Patients achieving complete metabolic response during immunotherapy have good prognosis, even in case of treatment discontinuation.
Dimitriou et al. [65]	2022	Nivolumab, pembrolizumab, ipilmumab	*n* = 104	EORTC, RECIST	A complete metabolic response before ICIs discontinuation, assessed according to EORTC, predicted long-term outcome and outperformed RECIST criteria as prognostic factor.
Ferdinandus et al. [66]	2022	Nivolumab, pembrolizumab, ipilmumab	*n* = 38	RECIST 1.1, EORTC/PERCIST for CMR definition	CMR to immunotherapy has a prognostic impact on long-term response after treatment discontinuation
Ellebaek et al. [67]	2022	PD-1 targeting immunotherpy	*n* = 140	CMR according to EORTC/PERCIST	The absence of ^18^F-FDG-avid lesions at the time of immunotherapy discontinuation is a powerful prognostic factor on long-term response.

## 4. Artificial Intelligence and Radiomics 

### 4.1. Basic Principles 

Artificial intelligence techniques (machine learning, deep learning, and radiomics) play a relevant role in nuclear medicine, in particular for the management of oncological and neurodegenerative disorders [69,70,71,72]. Radiomics can be defined as the high-throughput extraction of quantitative features from medical images (CT, MR and PET scans) to build classification and/or regression models. In recent years radiomics has received increasing attention as a means for computer-assisted diagnosis, prediction of survival and response to therapy. The classic radiomics workflow involves six sequential steps: acquisition, pre-processing, segmentation, feature extraction, post-processing and data analysis [69]. Feature extraction is the linchpin of the process. At present, there are two main classes of methods to feature extraction: the traditional (“hand-crafted”) ones and those based on deep learning. The hand-crafted ones, which include shape and texture features, are computed through mathematical functions designed by hand (‘feature engineering’); whereas in deep learning the features are implicitly generated by convolutional networks (CNN) trained on large datasets of images. 

Radiomics can, in principle, help obtain an in vivo classification of the disease, thus reducing the use of invasive diagnostic techniques, such as biopsy [73]. This is a valuable step towards precision medicine, targeted at the single patient.

### 4.2. Clinical Applications in the Field

The literature concerning the use of artificial intelligence techniques—for example radiomics—on ^18^F-FDG PET/CT imaging in the diagnosis of melanoma is relatively scarce. Guerrisi et al.’s [74] review spans 5 years, from 2014 until September 2019 and considers the following databases: MEDLINE/PubMed (National Center for Biotechnology Information, NCBI), EMBASE (Ovid) and the Cochrane Central Register of Controlled Trials (CENTRAL), Cochrane Library. The key search terms were: “neoplasms”, “melanoma”, “radiomics”, “texture analyses”, and “texture parameters”. The query returned a total of relevant 10 records, with most papers dating back to between 2017 and 2019 (*n* = 9) and one to 2015. The imaging modalities were: MRI (*n* = 4), CT (*n* = 4), PET/CT (*n* = 1) and PET only (*n* = 1). The sample size ranged from 21 to 80 patients per study, and the number of lesions from 23 to 483. These data indicate that the available literature on the use of PET scan and radiomics in the diagnosis of melanoma is still very limited.

Giesel et al. [75] retrospectively investigated PET/CT scans of 148 oncologic patients analyzing 1022 lymph nodes (LNs) from different tumors, specifically 327 LNs of 40 patients with lung cancer (LC), 224 LNs of 33 patients with malignant melanoma (MM), 217 LNs of 35 patients with gastroenteropancreatic neuroendocrine tumors (GEP NET) and 254 LNs of 40 patients with prostate cancer (PCA). A PET/CT scan was performed with different radiopharmaceuticals according to clinical indication, respectively, ^18^F-FDG for LC and MM, ^68^Ga-DOTATOC for GEP NET and ^68^Ga-labeled prostate-specific membrane antigen (^68^Ga-PSMA) for PCA. The aim of the study was to evaluate the correlation between SUVmax and semi-automated density measurements of LNs obtained by CT images of the PET/CT. Among the 1022 LNs examined, 331 were PET-positive (3 times the SUVmax of the blood pool), 86 PET-indeterminate (1–3 times the SUVmax of the blood pool) and 605 PET-negative (less than the SUVmax of the blood pool). Notably, PET-positive LNs showed significantly higher CT densities than PET-negative LNs regardless of the type of tumor. In conclusion, CT density of LNs in patients with LC, MM, GEP NET and PCA correlated with ^18^F-FDG, ^68^Ga-DOTATOC and ^68^Ga-PSMA uptake, respectively, and represent a potential additional parameter to discriminate between malignant and benign LNs. For PET-indeterminate LNs the authors conclude recommending the use of a 7.5 HU CT density threshold to differentiate between malignant and benign LN infiltration and 20 HU to exclude benign LN processes. The main limitation of this work, however, is the heterogeneity of the study population, which is not limited to melanoma.

Saadani et al. [76] conducted the first melanoma study, aiming at predicting BRAFV600 mutation status by using feature selection derived from ^18^F-FDG PET/CT scan. It shows that feature selection may contribute to better clarifying which descriptors are really useful for the statistical analysis. The work considered 70 unresectable stage III–IV melanoma patients undergoing baseline ^18^F-FDG PET/CT; these were assigned to the BRAFV600 (35 patients, 100 lesions) or BRAF wild-type group (35 patients, 79 lesions) according to the mutational status. For each lesion, 480 radiomics features related to morphology (*n* = 22), local intensity (*n* = 2), intensity-based statistics (*n* = 18), intensity-volume histogram (*n* = 6), intensity histogram (*n* = 24) texture (*n* = 408) and 4 conventional PET features (SUVmax, SUVmean, SUVpeak, and total lesion glycolysis) were extracted. The textural features included parameters from grey-level co-occurrence matrices (GLCM), grey-level run length matrices (GLRLM), grey-level size zone matrix (GLSZM), grey-level distance zone matrix (GLDZM), neighborhood grey-tone difference matrices (NGTDM), and neighbourhood grey-level dependence matrix (NGLDM). For feature selection, the authors considered 6 different approaches which they evaluated through 10-fold cross validation. For each patient, 1–10 target lesions were analyzed. The BRAFV600 and BRAF wild-type groups were not statistically different as for SUV metrics, MATV, TLG, longest diameter, or prior local intervention. Stratification per organ region of SUV metrics and TLG provided the same result. The best prediction model based on conventional PET features comprised all of them—that is, SUVmean, SUVmax, SUVpeak, TLG, and MATV. Radiomics analysis was carried out on 176 lesions (3 lesions from scans with a different voxel matrix were excluded). AUCs predicting the BRAFV600 mutation varied from 0.54 to 0.62 and were influenced by the feature selection method, being the best AUCs obtained by feature selection based on literature, a penalized binary logistic regression model, and random forest model. The authors conclude that BRAFV600 mutation status was neither associated nor predicted by conventional PET features, whereas radiomics features had predictive value (AUC 0.54–0.62). The authors also found that the feature selection methods had significant impact on the performance of the predictive models.

Reinert et al. [77] investigated the clinical and prognostic value of tumor volumetric parameters in patients with melanoma undergoing ^18^F-FDG-PET/CT as compared with serologic markers of tumor burden and inflammation. The authors evaluated 107 consecutive patients with malignant melanoma selected for potential surgical metastasectomy. Based on clinical evidence and PET/CT findings, 52 patients received surgical treatment, 32 systemic therapy, 2 palliative radiotherapy and1e isolated extremity perfusion, while the other 20 underwent watchful waiting. The volumetric PET parameters, whole-body Metabolic Tumor Volume (MTV), whole-body TLG and standard uptake value (SUV) peak, were quantified using 50%-isocontour volumes of interests (VOIs) and correlated with the following serologic parameters: lactate dehydrogenase (LDH), S-100 protein, C-reactive protein (CRP) and alkaline phosphatase (AP). The PET parameters were correlated with overall survival (OS) after PET/CT and a comparison in OS was performed among patients with or without metastases and increased or not-increased serologic parameters. LDH was strongly associated with MTV (rP = 0.73, *p* < 0.001) and TLG (rP = 0.62, *p* < 0.001), and moderately associated with SUVpeak (rP = 0.55, *p* < 0.001). S-100 protein showed moderate association with MTV (rP = 0.54, *p* < 0.001) and TLG (rP = 0.48, *p* < 0.001) and weak association with SUVpeak (rP = 0.42, *p* < 0.001). Furthermore, there was strong association between CRP and MTV (rP = 0.66, *p* < 0.001) and moderate to weak association between CRP and TLG (rP = 0.53, *p* < 0.001) and CRP and SUVpeak (rP = 0.45, *p* < 0.001). To discriminate subjects with or without metastases, receiver operating characteristic (ROC) analysis was performed showing an optimal cut-off value of 198 U/l for serum LDH (AUC 0.81, sensitivity 0.80, specificity 0.72). A multivariate analysis for OS was also carried out, determining that both MTV and TLG were strong independent prognostic factors. TLG, MTV and SUVpeak above patient median were associated with significantly decreased estimated OS compared with PET parameters below patient median (e.g., TLG: 37.1 ± 3.2 months vs. 55.9 ± 2.5 months, *p* < 0.001). On the other hand, high serum LDH and S-100 protein were associated with significantly low OS (36.5 ± 4.9 months and 37.9 ± 4.4 months) with respect to normal serum LDH (49.2 ± 2.4 months, *p* = 0.01) and normal S-100 protein (49.0 ± 2.5 months, *p* = 0.01). The authors conclude that tumor volumetric parameters from 18F-FDG-PET/CT can be useful prognostic biomarkers in advanced melanoma and that these are also associated with serologic tumor markers and inflammatory markers. The study has some limitations, in particular the retrospective nature; the incomplete availability of serologic parameters and survival data; and the lack of standardization in lesion segmentation. The latter, in particular, is critical: although the patients were examined in a single institution and MTV was measured using the same segmentation method, a 50% threshold was used for the isocontour VOIs instead of the 41% recommended by EANM, which can result in underestimated PET volumetric parameters.

Finally, Flaus et al. [78] aimed at predicting overall survival (OS) and progression-free survival (PFS) after one year of immunotherapy based on pre-treatment ^18^F-FDG PET scan data. To this end the authors retrospectively examined 56 patients with metastatic melanoma without prior systemic treatment. They considered 45 ^18^F-FDG PET-based radiomic features and retained the five features showing the strongest correlation with the patient’s outcome. Prediction models were obtained based on different machine learning classifiers, i.e., random forest (RF), neural network, naive Bayes, logistic regression, and support vector machine. The performance of the models was evaluated through cross-validation and receiver operating characteristic curves (ROC). The RF model achieved the best performance with AUC, sensitivity, and specificity, respectively, of 0.87 ± 0.1, 0.79 ± 0.11 and 0.95 ± 0.06 for OS; 0.9 ± 0.07, 0.88 ± 0.09 and 0.91 ± 0.08 for PFS (figures indicate 95% confidence intervals). The authors conclude that RF classifier, based on baseline ^18^F-FDG PET radiomic features might be helpful for predicting the survival status for melanoma patients one year after first line systemic immunotherapy.

## 5. Conclusions

^18^F-FDG PET/CT has shown great potential for the imaging and restaging of advanced MM; it greatly impacted patients’ management. In BRAF-mutated MM subjects, several PET-derived parameters (MTV, TLG) have been successfully applied for prognostic stratification prior to molecularly targeted therapy and to assess metabolic response during treatment. Of special note, PET/CT has been successfully implemented for monitoring MM patients under immunotherapy with ICIs: however, a jumble of different PET-interpreting criteria (PERCIMT, iPERCIST, imPERCIST5, etc.) has been proposed to correctly categorize possible paradoxical response linked to immunotherapy, namely pseudo-progression and hyper-progresion. Several efforts are being carried out to define the most appropriate PET-reading approach in MM patients submitted to immunotherapy, also specifically taking into account the adopted therapeutic regimen. 

Finally, the use of radiomics on nuclear medicine images can be a valuable tool towards personalized medicine in melanoma as well as other tumors. However, further studies—ideally larger, prospective, and possibly standardized—should be carried out in order to obtain robust and reproducible data.

## Figures and Tables

**Table 1:** Summary of main manuscripts focused on the role of ^18^F-FDG PET/CT for the imaging of advanced MM.

Authors	Year	Type of Study	Setting	Number of Patients	Comment
Gulec et al. [18]	2003	Retrospective, single-center	Impact on clinical management	*n* = 59	^18^F-FDG PET/CT detected additional lesions with respect to conventional imaging (total body CT) in the majority of patients and significantly impacted on therapeutic decision.
Querellou et al. [19]	2010	Retrospective, single-center	Additional value of lower limbs scan in MM patients	*n* = 122	In patients without known or suspected MM focuses on the lower limbs, additional scan did not add significant information or impacted on clinical managment.
Pfluger et al. [20]	2011	Retrospective, Single-center	Contribution of contrast-enhanced CT (ceCT) for PET/CT MM imaging	*n* = 50	PET/CT and PET/ceCT equally performed in advanced MM, especially in terms of specificity, therefore the use of conventional PET/CT (no-contrast media) modality is justified.
Bastiaannet et al. [21]	2012	Prospective, Single-center	Prognostic impact of PET-derived parameter, SUVmax	*n* = 80	In MM at stage IIIB, SUVmax measured on metastatic nodes before surgery can be used to patients prognostic stratification.
Holtkamp et al. [22]	2020	Prospective, Single-center	Staging and follow-up in patients with in-transit or satellite MM metastases	*n* = 25	PET/CT upstaged 4 out of 25 patients (16%) therefore leading to a change in clinical management. Furthermore, PET/CT detected the onset of distant metastases during follow-up in 10 cases within 6 months from diagnosis.
Klingestein et al. [23]	2010	Retrospective, Single-center	Restaging and follow-up of uveal melanoma	*n* = 11	PET/CT correctly identified metastases to liver, lungs, nodes and adrenal glands.
Cohen et al. [24]	2018	Retrospective, Single-center	Staging uveal melanoma	*n* = 108	PET/CT combined with abdominal ultrasonography resulted positive for metastases in 3 cases of uveal melanoma and identified a second primary malignancy in the 9% of the examined subjects.

**Table 3:** Main metabolic criteria applied for the assessment of response to immunotherapy in skin cancer.

Authors	Year	Criteria	CMR	PMR	PMD	SMD
Wahl et al. [47]	2009	PERCIST	Complete regression of all ^18^F-FDG-avid sites	SULpeak reduction in at least 30% in the target lesions	Increase in SULpeak of at least 30% or new lesions	None of the previously mentioned conditions
Sachpekidis et al. [48]	2015	EORTC	Complete regression of all ^18^F-FDG-avid sites	Minimum reduction of ±15–25% in SUV after the 1st cycle of chemotherapy, and >25% after more than one cycle	Increased SUVmax of ≥25% or appearance of new lesions	None of the previously mentioned conditions
Anwar et al. [49]	2018	PERCIMT	No new lesions (Clinical Benefit)	No new lesions (Clinical Benefit)	>4 new lesions with functional DM <1 cm, or three new lesions with functional diameter >1 cm or two new lesions with functional diameter >1.5 cm	None of the previously mentioned conditions
Goldfarb et al. [50] Filippi et al. [51]	2019 2022	iPERCIST	Complete regression of all ^18^F-FDG-avid sites	SULpeak reduction of at least 30% in the target lesions	Increase in SULpeak of at least 30% or new lesions (unconfirmed progressive disease/UPMD), needing confirmation (cPMD) with a further scan after 4–8 weeks.	None of the previously mentioned conditions
Ito et al. [52]	2019	Immunotherapy-modified PERCIST (imPERCIST5)	Complete regression of all ^18^F-FDG-avid sites	Sum of SULpeak decreased by at least 30%	Increase in the sum of SULpeak by at least 30%	None of the previously mentioned conditions
